# Dynamic Clustering of Gene Expression

**DOI:** 10.5402/2012/537217

**Published:** 2012-10-16

**Authors:** Lingling An, R. W. Doerge

**Affiliations:** ^1^Department of Agricultural and Biosystems Engineering, University of Arizona, Tucson, AZ 85721, USA; ^2^Department of Statistics, Purdue University, West Lafayette, IN 47907, USA

## Abstract

It is well accepted that genes are simultaneously involved in multiple biological processes and that genes are coordinated over the duration of such events. Unfortunately, clustering methodologies that group genes for the purpose of novel gene discovery fail to acknowledge the dynamic nature of biological processes and provide static clusters, even when the expression of genes is assessed across time or developmental stages. By taking advantage of techniques and theories from time frequency analysis, periodic gene expression profiles are dynamically clustered based on the assumption that different spectral frequencies characterize different biological processes. A two-step cluster validation approach is proposed to statistically estimate both the optimal number of clusters and to distinguish significant clusters from noise. The resulting clusters reveal coordinated coexpressed genes. This novel dynamic clustering approach has broad applicability to a vast range of sequential data scenarios where the order of the series is of interest.

## 1. Introduction 

 Microarray and next-generation sequencing (RNA-seq) technologies enable researchers to study any genomewide transcriptome at coordinated and varying stages. Since biological processes are time varying [1], they may be best described by time series gene expression rather than by a static gene expression analysis. Acknowledging the nature of genes that are involved in dynamic biological processes (e.g., developmental processes, mechanisms of cell cycle regulation, etc.) has potential to provide insight into the complex associations between genes that are involved. 

 Functional discovery is a common goal of clustering gene expression data. In fact, the functionality of genes can be inferred if their expression patterns, or profiles, are similar to genes of known function. There are published clustering methods that include into the analysis the duration of the experimental stages, or the staged dependence structure of gene expression. The results from these approaches are certainly more informative and realistic than groupings that are gained from static clustering methods (i.e., clustering at a single-staged experimental point), but their results are limited in interpretation. The seminal work from Luan and Li [2] is a good example of a clustering application that takes the time dependent nature of genes into account. More realistic, though, is the fact that some biological processes typically start and end at identifiable stages, or time points, and that the genes in a process may be dynamically regulated at different stages of the biological process [3]. In other words, genes can be coregulated over a finite series of points (i.e., only a portion of points represent the total when the transcriptome is being sampled). 

A variety of subspace clustering methodologies have attempted to address the time-dependent nature of transcriptome experiments through biclustering [4], or plaid models [5]. Although these bicluster (i.e., clusters obtained by any subspace clustering method are referred to as biclusters from this point forward) approaches are popular, they have limitations. Namely, they restrict subspace clusters to consecutive time points [6–9]. For example, Madeira and Oliveira [8] discretized real-valued gene expression data as upregulated, downregulated, and unchanged according to the slope of expression change from one time point to the next. They then rely on string processing techniques to develop an algorithm that identifies contiguous column coherent biclusters. Alternatively, Zhang et al. [9] alter original expression data by deleting and inserting border time points, and then use an algorithm based on a mean squared residue score to cluster the modified expression data. 

 We are motivated by the fact that the genes involved in the biclusters that are obtained by [8, 9] have the same starting and ending time point(s). Even though it is well known that time lags exist for many genes that are involved in the same biological process and that genes with the same function may give rise to unique expression patterns/profiles, to our knowledge this information has not been incorporated into any statistical approach for clustering. Ji and Tan [6] focus on extracting time-lagged gene clusters known as *q*-clusters, where *q* is the time length of a bicluster (i.e., the number of consecutive time points in the bicluster), that can have different time lengths, but genes in the same cluster must have the same durations over time, even though time lags exist among the genes. Song et al. [7] proposed to use a wavelet-based cluster method to detect time shift/delay situation. To our knowledge, none of the current or existing subspace clustering methodologies is able to provide biclusters that are varying in their duration of time length.

**Figure 1:**
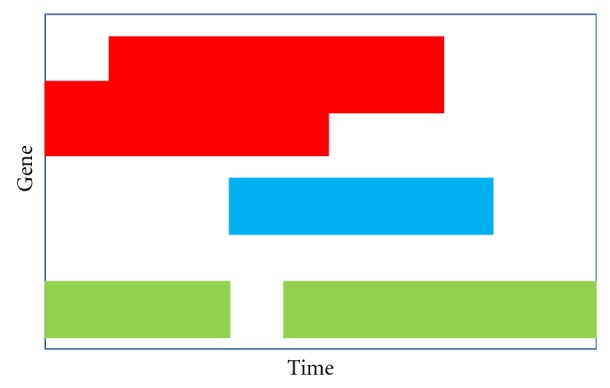
Three (red, blue, green) hypothetical gene clusters across time. The specific genes in the red cluster is varying, and of different duration, while the genes in the green cluster reenter the cluster after a time of not being in the cluster, and all of the genes in the blue cluster enter and leave the process at the same time.

 We know that standard exploratory clustering methods are useful for grouping items that behave in a similar fashion. However, when these standard approaches are applied to experiments that evaluate the transcriptome over coordinated experimental stages, they fail to acknowledge the dynamic nature of such processes. As such, this work focuses on the dynamic and nonconsistent nature of gene activity (Figure 1). Although presented here in the context of coordinated transcriptome data, this novel dynamic clustering approach is applicable to a vast range of sequential data scenarios where the order of the series is of interest [10].

## 2. Methods

 One important aspect of periodic (gene expression) profiles is spectral frequency. Specifically, there may be discontinuities (e.g., a gene may enter or exit a biological process at any time point), or more generally, a time-varying spectral frequency in nonstationary or piecewise stationary time series (i.e., signal) that can be studied using techniques and theories from signal decomposition [11]. “Time series” and “signal” are used exchangeable from this point forward. Once decomposed, the components can be quantified based on their constrained coherency (CoCo, details in the Section 2.2) and gathered via an agglomerative hierarchical clustering that involves determining the number of clusters and separating signal from noise. 

### 2.1. Time Frequency Analysis

 For a nonstationary time series, time-frequency analysis is usually employed to map one-dimensional time series onto a two-dimensional time-frequency domain so that both the frequency and time information can be considered [12, 13]. Addison [14] justifies the continuous wavelet transformation (CWT) based time-frequency analysis and states that it has many benefits over other time-frequency representations of multiple-component signals (i.e., signals that contain multiple frequencies) or signals with discontinuities in frequency [13]. We assume that even though a gene may be expressed multiple times in a time series, it may also be involved in multiple biological processes, and therefore multiple spectral components need to be considered [15]. Toward this end, we employ CWT to decompose gene expression signals, *s*(*t*):

(1) $$\begin{array}{c} W\left(b,a\right)=\frac{1}{a}\int s\left(t\right)\varphi \left(\frac{t-b}{a}\right)dt, \end{array}$$

where *φ*(*t*) is the mother wavelet, *a* is the scale parameter, *b* is the shift parameter, and the Morlet wavelet [16] is employed. The significant frequency values and their associated starting and ending time points for an expression signal, *s*(*t*) = (*s*_1_, *s*_2_, …, *s*_*N*_), are determined as follows.

1. Perform a continuous wavelet transformation (CWT) on the time series *s*(*t*).
2. Generate a white noise time series *w*(*t*) = (*w*_1_, *w*_2_, …, *w*_*N*_), where *w*_*i*_ *'*s are distributed as *N*(0,*σ* ^2^), where *σ* ^2^ is the variance of the time series *s*(*t*).
3. Perform a CWT on the white noise time series from Step  2 and obtain the time-frequency representation.
4. Repeat Steps 2-3 *M* times (usually, *M* ≥ 1000). For each frequency *f* at each time point *t*, the 95th percentile of *M* modulus serves as the threshold. The threshold surface is constructed by connecting all thresholds across both frequency and time.

Let *W*_0_(*t*, *f*) represent the threshold surface calculated from white noise information, and let *W*(*t*, *f*) represent the CWT outcome of the original gene expression signal *s*(*t*). Determining the significant components whose CWT values are above the threshold surface is equivalent to identifying the components from *W*′:

(2) $$\begin{array}{c} W^{\prime }\left(t,f\right)=\max\;\left\{0,W\left(t,f\right)-W_{0}\left(t,f\right)\right\}, \end{array}$$

where *t* represents time and *f* denotes frequency. It is worth noting that the meaningful component on the sliced CWT has some width in the frequency domain, and that a signal may contain several significant components. A “Crazy Climber” ridge extraction algorithm is employed [12] to extract significant frequencies and their related starting and ending time points. 

### 2.2. Similarity Measurement

 The actual clustering (of genes) requires a similarity metric that measures the pairwise relationship between genes via their decomposed components. Since frequency is of interest, a coherency [17] is used. For two signals *x* and *y*, coherency is

(3) $$\begin{array}{c} C_{xy}\left(f\right)=\frac{P_{xy}^{2}\left(f\right)}{P_{xx}\left(f\right)P_{yy}\left(f\right)}, \end{array}$$

where *P*_*xx*_(*f*) and *P*_*yy*_(*f*) are the respective power spectral densities of the two signals and *P*_*xy*_(*f*) is their cross-spectral density. Typically when calculating the coherency function between two signals, the lengths of the signals are the same. However, since the component signals may differ in length, the median length for all frequencies is used to represent a uniform time length. Further, since coherency is a function of frequency, its range is from zero to half of the sampling frequency of a discrete signal (i.e., a continuous signal measured at discrete time points; [18]). In general, the sampling frequency, or sampling rate, is the number of time points per second as measured in Hertz. Given two signals with frequencies *f*_*x*_ and *f*_*y*_, we use the average of the coherency function in the interval [*f*_*x*_, *f*_*y*_] to represent the coherency similarity and refer to it as “coherency” for simplicity.

**Figure 2:**
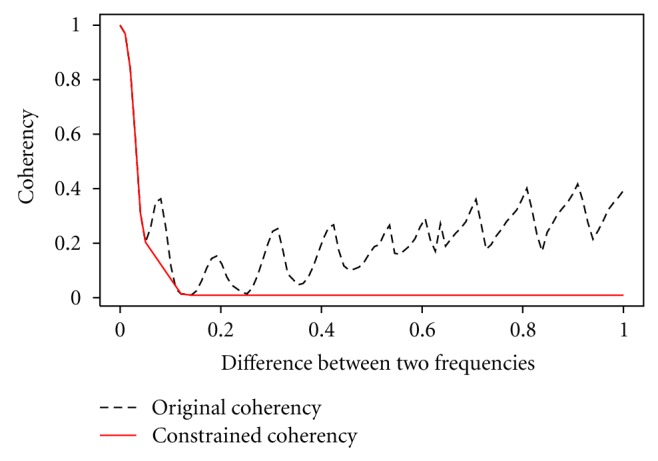
The nonmonotonic pattern of frequency differences between two signals requires a modification to the coherency. A constrained coherency (CoCo) identifies the valleys of the original curve as nonincreasing along the frequency differences and provides a monotonic representation of coherency. Illustration taken from An and Doerge [20].

Although it is expected that coherency will decrease as the difference between two frequencies (from two signals) increases, there are situations (Figure 2) when a nonmonotonic coherency pattern identifiable from a coherency plot exists. To ensure monotonicity, we provide a modified coherency that acknowledges that the valleys of the coherency curves are nonincreasing along the frequency differences when we construct a representative curve that is monotonic in frequency difference. Although many other similarity measures can be employed (e.g., Brownian Distance measurement [19]), our modification (CoCo) is data-driven and provides a constrained coherency that serves as a similarity measurement for clustering (Figure 2).

### 2.3. Clustering

 We rely on agglomerative hierarchical clustering coupled with Ward linkage [21] for clustering. Although the Ward linkage finds compact and homogenous clusters by minimizing the variance of objects, there are two issues that we need to address: determining the number of clusters and differentiating meaningful clusters from noise clusters (i.e., clusters containing only noise). 

### 2.4. Cluster Validation

#### 2.4.1. Number of Clusters

 A variety of approaches have been suggested for determining the number of clusters [22–24]. One well-known approach involves finding the “elbow” (or, change point) of an error curve [25]. Unfortunately, most work that relies on the elbow lacks statistical justification [26]. Although others have attempted to compare the error curve of the original data to the error curve of the data generated from a null reference distribution (i.e., uniform distribution) by employing the Gap statistic [27], this approach is not applicable to overlapping clusters, nor is it appropriate for noisy data [24, 25]. 

 We approach the issue of determining the number of clusters by globally evaluating the merge distance plot, which is represented by the height of joint nodes in a cluster tree. A null reference distribution is able to provide a merge-distance threshold that can be compared to the original merge distance. Specifically, a minimal convex set, or a convex hull, is formed based on a set of convex combinations of all points of interest [28]. The merge distance is obtained by performing clustering on data generated from a uniform distribution from the convex hull. Munneke et al. [29] used a convex hull to generate randomly distributed gene expression values so that distinctions between clusters arise with statistical confidence. Here, a convex hull is employed to assess the statistical significance of the merge distance of hierarchical clusters. By evaluating the merge distance globally, the number of clusters can be determined. 

 For a set *X* containing *n* objects, {*x*_1_, …, *x*_*n*_}, the convex hull of *X* is

(4) $$\begin{array}{c} H\left(X\right)=\left\{\sum_{i=1}^{n}\alpha_{i}x_{i}\;|\;x_{i}\in X,\alpha_{i}\geq 0,\sum_{i=1}^{n}\alpha_{i}=1\right\}, \end{array}$$

where *x*_*i*_ can be one or more dimensions. For time series gene expression data, *n* is the total number of decomposed components and the *x*_*i*_'s are the component frequencies. The following steps are used to determine the number of clusters.

1. Perform hierarchical clustering with Ward linkage and CoCo similarity on the original data (*x*_*i*_) containing *n* points and obtain the merge distance set *M*_0_ = (*d*_2_, …, *d*_*n*_).
2. Randomly choose *n* objects from the uniform distribution [min(*x*_*i*_), max(*x*_*i*_)].
3. Perform hierarchical clustering on the random data set from Step (2), and obtain a new merge distance set.
4. Repeat Steps (2)-(3) *M* times (usually *M* ≥ 1000). For each possible number of clusters (*k*, *k* = 2,…, *n*) the 95th percentile of *M* merge distances, *d*_k_^*^, serves as a threshold and the 95% threshold curve is constructed as *M* ^*^ = (*d*_2_^*^, …, *d*_n_^*^).
5. Compare *M*_0_ with *M* ^*^; the largest *k* which satisfies *d*_*k*_ > *d*_k_^*^ is the optimal number of clusters *k*_0_.

Since some gene expression data can be quite noisy, an additional step in cluster validation necessitates differentiating the noise cluster from meaningful (i.e., significant) clusters. 

#### 2.4.2. Significant Clusters

 A noise cluster differs from statistically significant clusters in terms of compactness and separation. The objects in a noise cluster are scattered, while the objects in a statistically significant cluster, which are similar to the tight clusters in [30], are dense. Here, the silhouette metric [31], a measure of tightness and separation of clusters, is used to assess the level of statistical significance of clusters. For each object *i*, its silhouette width is defined as

(5) $$\begin{array}{c} s\left(i\right)=\frac{b\left(i\right)-a\left(i\right)}{\max\;\;\left\{a\left(i\right),b\left(i\right)\right\}}, \end{array}$$

where *a*(*i*) is the average dissimilarity of object *i* to other objects in the same cluster and *b*(*i*) is the average dissimilarity of object *i* to objects in its nearest neighbor cluster. The range of the silhouette width is [−1, 1]. The average silhouette width of objects in a cluster represents the quality of the cluster in terms of compactness and separation. The average silhouette of a noise cluster (if it exists) should be low, while for statistically significant clusters it should be high. 

 When evaluating the silhouettes for *k*_0_ clusters, a uniform reference distribution is employed to generate independently located objects upon which the hierarchical clustering operates. The silhouettes are obtained for the reference data for the same cluster number *k*_0_ as follows [20].

1. For *k*_0_ clusters from the original data, compute their silhouettes.
2. Randomly choose *n* objects from the uniform distribution on the convex hull of the frequencies of the original data.
3. Perform hierarchical clustering on data from Step (2), choose *k*_0_ clusters and obtain their silhouettes.
4. Repeat Steps (2)-(3) *M* times (usually *M* ≥ 1000) and obtain *M* sets of *k*_0_ silhouettes. For each of *k*_0_ silhouettes of the original data, calculate its *P* value from *M* ^*^ *k*_0_ values. Cluster significance is represented by its *P*-value.
5. The significance level is *α*. A cluster is significant if its *P* value < *α*; otherwise, it is noise.

### 2.5. Dynamic Cluster

 Up to this point, we have described a two-step cluster validation that provides the number of clusters and differentiates significant clusters from a noise cluster. Since a gene's expression over time can be described in terms of frequencies and then decomposed into components that each may have unique start and stop points, the time-dependent structure of the data is retained, and the concept of a dynamic cluster evolves naturally.

In anticipation of assessing the performance of the proposed dynamic clustering algorithm via simulation, we realize two further issues. First, to our knowledge, there are no clustering approaches, which provide gene sets at different (time) points. This makes comparing our approach with existing approaches fruitless. Second, because we work from simulated data, we need a metric to compare the clusters that result from dynamic clustering with the cluster-scenario from which they were simulated. To address these issues, we develop a discovery index.

### 2.6. Discovery Index

 The proposed cluster validation algorithm objectively evaluates the quality of clustering results using information from the data (i.e., silhouette width). When prior information (e.g., pathway, etc.) is available, obviously it contributes even more information upon which to base cluster validation. Fortunately, this is exactly the situation for time series gene expression with time-varying frequencies; information on both true cluster membership and true time duration for genes in clusters is available. Since traditional criteria [32, 33] only involve the true cluster membership, we provide a new criterion that takes into account both time information and cluster characteristics.

 Assume an estimated cluster *E* obtained via a clustering method represents the true class *C* such that some genes in *E* differ from genes in class *C* in terms of the gene identification number or the gene time information. For each gene in either true class *C*, or estimated cluster *E*, consider the true time interval and estimated time interval. The discovery index for gene *g* in class *j* or cluster *j* is defined as

(6) $$\begin{array}{c} D_{gj}=\frac{P_{gj}\cap Q_{gj}}{P_{gj}\cup Q_{gj}}, \end{array}$$

where *P*_*gj*_ denotes the true time interval and *Q*_*gj*_ denotes the estimated time interval. By this definition, 0 ≤ *D*_*gj*_ ≤ 1. Specifically, when gene *g* appears in the class but is not detected in the corresponding cluster we have *D*_*gj*_ = 0 since *Q*_*gj*_ = 0; similarly, when it is detected in the cluster but is not in the corresponding class we get *D*_*gj*_ = 0 due to *P*_*gj*_ = 0. For a class it is possible to define its cluster by using all pairwise comparisons and a specified (small) cut-off. We can find the closest cluster (i.e., estimate) for each class. Specifically, if the distance between a class and its closest cluster (i.e., the difference between two frequency values) is less than the predefined cutoff, then the cluster will be called its estimate. At times not all of the classes will be detected, or some cluster may be superfluous since it may not have a matching class. In these situations, the discovery indices for the genes in either that class or that cluster are all zero. Finally, the overall discovery index for the genes across the corresponding clusters and classes is

(7) $$\begin{array}{c} D=\frac{\sum_{j=1}^{J}\sum_{g=1}^{G}D_{gj}}{\sum_{j=1}^{J}O_{j}}, \end{array}$$

where *G* the number of genes, *J* is the maximum of the number of clusters and the number of classes, *O*_*j*_ is the number of genes in the *j*th cluster or class, and *D*_*gj*_ is the discovery index for gene *g* in the *j*th cluster or class. Clearly, ∑_*g*=1_^*G*^ *D*_*gj*_ ≤ *O*_*j*_, so 0 ≤ *D* ≤ 1. The discovery index evaluates the value of combining clustering and signal decomposition.

## 3. Simulation Studies

 Because the proposed approach includes two subprocedures, namely, time-frequency decomposition and clustering, and because the result of the first subprocedure impacts the results of the second subprocedure, a power study will be conducted on these two subprocedures separately. The power of the signal decomposition is investigated relative to the noise level, frequency level, difference between frequencies of components in a gene signal, and time lengths of components. Interestingly, gene expression contains various features, for example, some genes may have one component with a certain frequency; some genes may have two components with other frequencies; others may have more than two components with different frequencies. We acknowledge that investigating the performance of the signal decomposition, and the proposed clustering method, for these types of data is challenging simply because it is difficult to summarize the distribution of the frequencies for multiple components from multiple genes. Furthermore, the time durations of the components affect statistical power.

### 3.1. Time-Frequency Analysis

 Signal decomposition is influenced by many parameters/factors, including frequency, frequency difference (for multiple component signals), time length of signal, ratio of the amplitude of the components, and noise level. Because of the limitation of displaying multiple factor effects simultaneously, a power study of signal decomposition on two-component signals is performed for investigating the effect of frequency, frequency difference, amplitude ratio, and noise. Additional simulation studies can be found in An's paper [34]. In general, the two-component signals are simulated from

(8) s(t)=cos (2πft+φ1)+Acos (2π(f+Δf)t+φ2)+  noiselevel∗N(0,1),

where *t* is time from 0 to 10 seconds,* f* represents spectral frequency that varies from 0.1 Hz to 1.0 Hz, Δ*f* denotes the frequency difference between two components (from 0.1 Hz to 1.0 Hz), *A* represents the amplitude ratio between two components (0.5, 0.2, 1.0, 2.0, 5.0; the amplitude of the first component is 1, as a baseline), and *φ*_1_ and *φ*_2_ are the phase shifts that are randomly chosen from [0, 2*π*]. The noise level varies from 0 to 1.0, in increments of 0.10. In spectral analysis, particularly in fast Fourier transformations, the length of a signal is usually a power of two. We consider 64 time points that equally partition the sample space. 

 The power of decomposing each gene expression time series is defined as

(9) $$\begin{array}{c} \frac{\sum_{k=1}^{c}w_{k}}{\sum_{i=1}^{q}u_{i}+\sum_{j=1}^{m}v_{j}-\sum_{k=1}^{c}w_{k}}, \end{array}$$

where ∑_*i*=1_^*q*^ *u*_*i*_ is the total time duration of the *q* true components (*q* = 2 for the two-component signal decomposition), ∑_*j*=1_^*m*^ *v*_*j*_ is the total time duration of the *m* estimated components, and ∑_*k*=1_^*c*^ *w*_*k*_ is the total time duration of the *c*overlap components between the true and estimated components. For each parameter combination, the overall power of the signal decomposition is the average power for decomposing 1000 signals.

**Figure 3:**
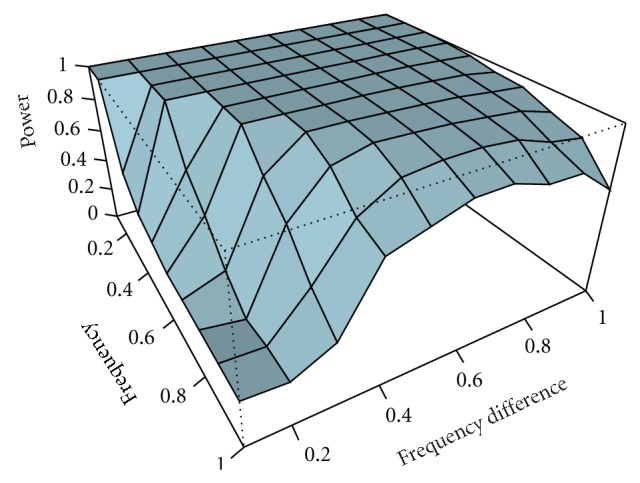
The decomposition power of CWT for two-component signals without noise. The two components, both with the same amplitude (i.e., ratio 1), have different frequencies.

Figure 3 illustrates the power study of decomposing two-component signals with amplitude ratio 1.0 without noise. A larger difference between two frequencies results in greater power, and a lower frequency is more likely to be detected than a higher frequency. Similar conclusions are obtained for decomposing signals with different amplitude ratios and different noise levels (plots not shown). The effect of time duration is also investigated. Components with longer time duration are more likely to be detected than those having short time duration (plots not shown). 

### 3.2. Dynamic Clustering

 The performance of the dynamic clustering approach coupled with the proposed validation method is investigated using the discovery index, which reflects the effect of both the signal decomposition and the clustering. It is a considerable challenge to display the discovery index for a set of data across all possible combinations of parameters. Fortunately, it is possible to assess the effect due to noise in the discovery index while holding the other parameter settings fixed. The performance of the dynamic clustering is assessed via fixed parameters over increasing noise. Further, the effect of time, when other parameter settings are fixed, is also assessed. Relying on the previous simulation, we use 140 simulated genes to illustrate the dynamic nature of the simulated time series. 

#### 3.2.1. Power Study: Noise Effect

 Time series expression data for 140 genes in three groups are simulated as follows:

(10) group  1=cos  (2π∗0.1t+φ1)+cos  (2π∗0.8t+φ2)+noiselevel∗N(0,1),group  2=cos (2π∗0.4t+φ3)+cos (2π∗0.8t+φ4)+  noiselevel∗N(0,1),group  3=cos (2π∗0.1t+φ5)+cos (2π∗0.4t+φ6)+  noiselevel∗N(0,1).

The time *t* and the phase shifts *φ*_*j*_  (*j* = 1,…, 6) are simulated as in the previous power study. There are 20, 40, and 80 genes in groups 1, 2, and 3, respectively. Each gene expression profile contains two components whose frequencies are 0.1 Hz, 0.4 Hz, and 0.8 Hz. Each pair of genes from different groups shares one common component. Sixty-four time points equally partition the sample space [0, 10]. Noise level varies at 0.5, 1, and 1.5.

**Figure 4:**
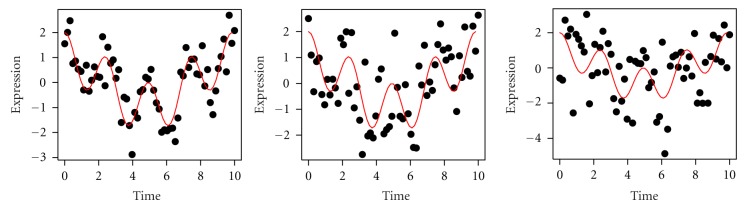
A single simulated gene expression profile with components 0.1 Hz and 0.4 Hz at three different noise levels: 0.5, 1, and 1.5 (from left to right). The red line represents each profile without noise.

The discovery index is calculated for each scenario, and the average discovery index-calculated for each of the 1000 simulated data sets (Table 1).  Figure 4 illustrates single gene expression profiles with two components, 0.1 and 0.4 Hz, at three different noise levels (0.5, 1, and 1.5). Interestingly, the discovery index is very high even though the data are moderately noisy (i.e., noise level 1.0 and the energy ratio from signal and noise is 1 : 1). Dynamic clustering using the proposed similarity metric and validation methods is able to capture meaningful information from relatively noisy data. 

#### 3.2.2. Dynamic Clustering for Various Time Lengths

 The components in the previous simulation are simulated (3) under varying times (0 to 10), across the entire time interval. Since some genes may belong to a cluster in only a portion of a time interval (simply because of noise), dynamic clusters are demonstrated using simulated data where time intervals for some components are only a portion of entire time interval. One hundred and forty genes are simulated as follows:

(11) group  1=cos (2π∗0.1t+φ1)+N(0,0.5), 0≤t<5=cos (2π∗0.1t+φ2)+cos (2π∗0.8t+φ3)+N(0,0.5), 5≤t≤10,group  2=cos (2π∗0.4t+φ4)+cos (2π∗0.8t+φ5)+N(0,0.5), 0≤t<5=cos (2π∗0.4t+φ6)+N(0,0.5), 5≤t≤10,group  3=cos (2π∗0.1t+φ7)+N(0,0.5), 0≤t<5=cos (2π∗0.1t+φ8)+cos (2π∗0.4t+φ9)+N(0,0.5), 5≤t≤10.

**Figure 5:**
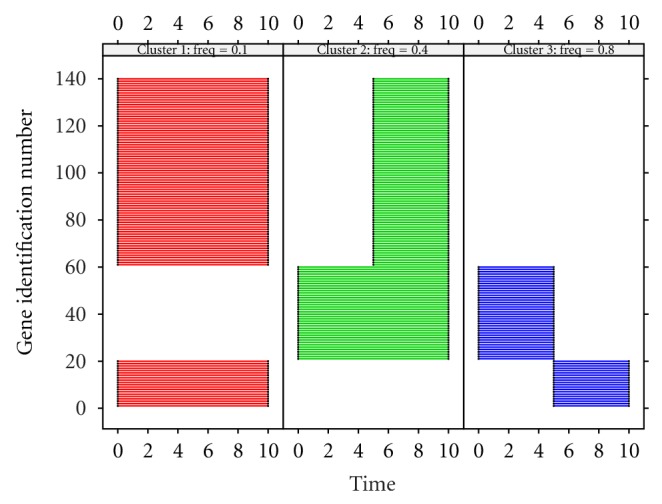
Panel plot for the true three clusters of 140 genes simulated using (8). Each segment represents a component (gene id on the *y*-axis) with the starting and ending time points detected via signal decomposition. The frequency value in each cluster is given at the top of each panel.

The true clusters (i.e., the clusters from which the 140 genes are simulated) are illustrated in Figure 5 where different colors represent different clusters. In each panel, the segmentation represents the starting and ending time points of the corresponding genes involved in that cluster. The cluster characteristics are represented by the component frequencies, 0.1, 0.4, and 0.8 Hz, and are listed on the top of each panel. 

**Figure 6:**
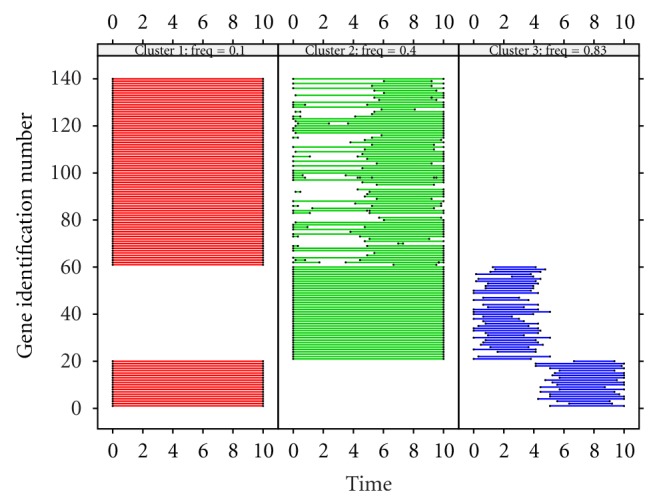
Panel plot for the estimated three clusters from 140 genes simulated using (8). Each segment represents a component (gene id on the *y*-axis) with the starting and ending time points detected via signal decomposition. The estimated frequency value for each cluster is shown at the top of each panel.

 Three significant clusters are detected by cluster validation. The dynamic property of the clusters is displayed in Figure 6 and is visibly comparable to Figure 5 (simulation setting). In the (red) cluster with frequency 0.1 Hz, all genes from group 1 and group 3 are involved during the whole time interval. For the (green) cluster with frequency 0.4 Hz, all genes from group 2 appear in the entire interval. In this cluster, some genes from group 3 remain in the second half of the time interval and some genes are in the entire time interval. For the third (blue) cluster with frequency of 0.83 Hz, most genes in group 1 are active in the second half interval and most genes in group 2 are active in the first half interval. Figure 6 reveals that components with a lower frequency, and of long duration, are unlikely to be affected by noise during the decomposition. Interestingly, the edge effect of time series is involved in the results (Figure 6). Specifically, the starting or ending time points for the genes in the third (blue) cluster may not be accurately estimated, yet they can be estimated precisely for the genes of a long duration (e.g., the genes in the first (red) cluster and genes from the second group in the second (green) cluster). 

 Although we relied on the 140 genes from our earlier simulation to demonstrate the performance of the proposed method, it is worth noting that performance improves as the number of genes increases. Specifically, increasing gene number allows our algorithm to more accurately identify the noise cluster, thus separating the gene cluster(s) of interest more precisely. While our simulation studies demonstrate that our approach is able to both capture meaningful signals from very noisy data and group them very well, we cannot compare our method with existing methods [36] simply because no information about time is contained in the clusters obtained by other methods.

## 4. Real Data Application

 Many microarray experiments have been conducted for the purpose of understanding complex dynamic biological processes and gene function of cell cycle (e.g., yeast, human fibroblasts, human cancer cell lines, and *Plasmodium falciparum*) [37–40]. Applicable here is the fact that it is essential cell-cycle genes exhibit periodic expression over time. Dynamic clustering is applied to cell-cycle *Plasmodium falciparum* (known to cause malaria in humans) expression data from [35]. 

### 4.1. *Plasmodium falciparum*

 Between the mosquito vector and human host, *Plasmodium falciparum* has a complex life cycle. Its genome is sequenced and has over 5,000 cell-cycle genes; 530 of them are annotated into 14 functional groups [35]. A more complete understanding of the life cycle and gene regulation will provide the foundation for drug and vaccine development, for example, shortening its life cycle may control transmission. Most of studies on the *P. falciparum* data focus on either detecting periodic genes [41, 42] or static clustering [43, 44]. Dynamic clustering is applied to the 530 gene representations measured at 46 time points spanning 48 hours during the Intraerythrocytic Developmental Cycle (IDC) with 1 hour time resolution for the HB3 strain. The data are downloaded from http://dx.doi.org/10.1371/journal.pbio.0000005.st002. The missing data (for time point 23 and 29) are imputed by the *k*-nearest neighbor algorithm [45] with *k* = 12. 

### 4.2. Results

**Figure 7:**
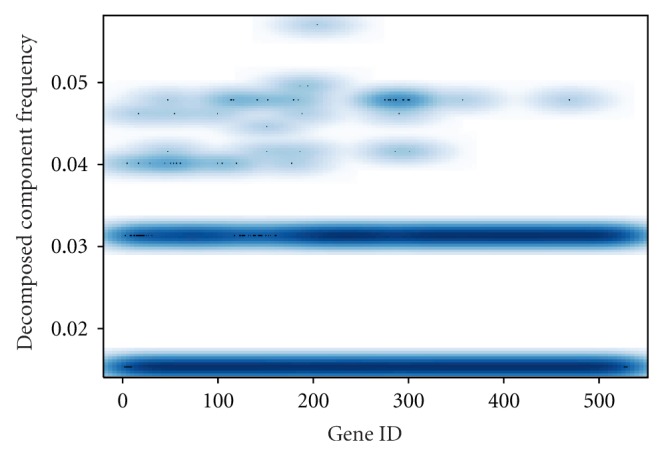
Smoothed scatter plot of decomposed frequency (centralized) of 1,019 components for 530 *P. falciparum* genes [35]. The dark blue area represents the high density of the points.

**Figure 8:**
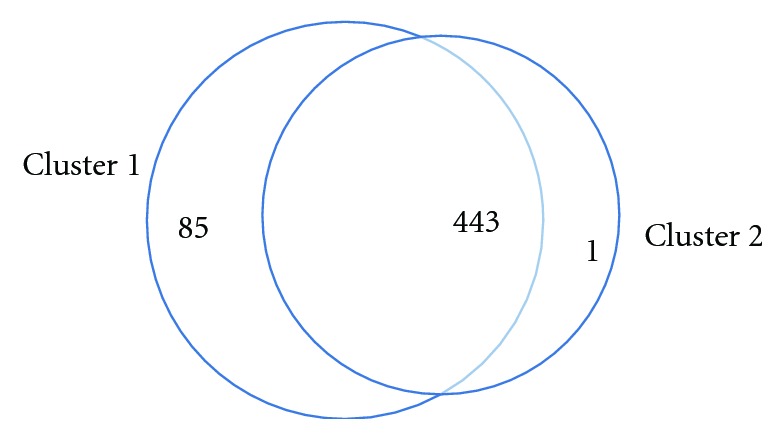
Distribution of the gene counts in the two significant clusters that result from an application of dynamic clustering to *P. falciparum* data from Bozdech et al. [35].

 Using the continuous wavelet transformation and ridge extraction, 530 time series gene expression profiles are decomposed into a set of 1,019 component signals whose frequencies are centralized for the purpose of calculation. Hierarchical clustering using CoCo similarity is employed. Two significant clusters and one noise cluster are detected (Figure 7). The number of genes in each (and between) significant cluster is summarized in Figure 8. 

**Figure 9:**
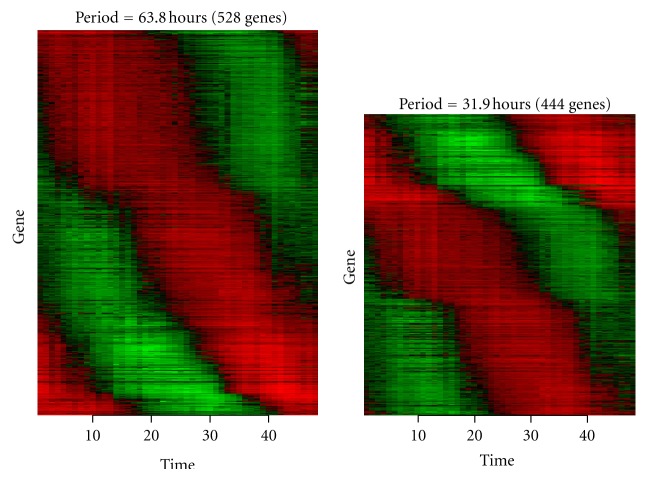
Expression profiles for the 530 *P. falciparum* genes [35] in the two different clusters that are found by dynamic clustering. The number of genes and their average period is at the top of each plot. The *x*-axis represents time (from 1 to 48 hours) and the *y*-axis represents genes that are ordered by their phases (from the signal decomposition). Red represents high expression values and green represents low expression values.

In the signal decomposition, the phase or phase shift can be obtained for each component.  Figure 9 summarizes genes that are ordered by the phase (shift) of the corresponding component. Red represents high values and green denotes low values. The cluster characteristic is represented by period (reciprocal of frequency). 444 genes are in the cluster with period of 31.9 hours and 528 genes are in the cluster with period of 63.8 hours. The periods of 31.9 hours and 63.8 hours are equivalent to1.5 cycles and 0.75 cycles, respectively. These are consistent with the findings in the original research [35]. Specifically, that the majority of the periodic gene profiles exhibit an overall expression period of 0.75~1.5 cycles in 48 hour interval. 

**Figure 10:**
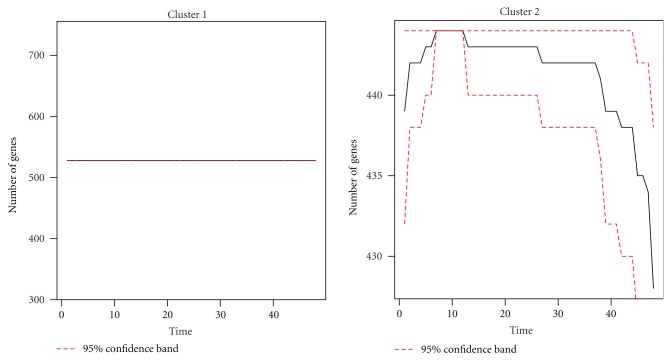
The number of genes varies across time in each of the two clusters found from dynamic clustering of 530 *P. falciparum* genes. The red dashed lines are the 95% bootstrap confidence band.

Since the components contain time information (i.e., starting and ending time points), the number of genes in a cluster may vary across time. The dynamic property of clusters from the *P. falciparum* data can be summarized in terms of number of genes at each time point (Figure 10). A nonparametric bootstrap [46] is employed to calculate the 95% confidence interval for the statistic. Hence the 95% confidence band for the curve of the number of genes is constructed by calculating the confidence intervals across all time points. 

As illustrated in Figure 10, the number of genes in Cluster 2 varies with time while the number of genes in Cluster 1 (i.e., the ones with longer period) remains constant. This confirms the findings from the simulation study that genes with lower spectral frequency are more likely to be detected than those with higher frequency. Apparently, multiple periods in *P. falciparum* data have not been studied (i.e., no published results); therefore, the expression pattern in Figure 10 may warrant further investigation.

### 4.3. Gene Ontology Analysis of the Clustered Genes

We employed the web-accessible programs DAVID (Database for Annotation, Visualization and Integrated Discovery [47, 48]) and PlasmoDB (Plasmodium Genomics Resource [49]) for the gene ontology (GO) analysis. In DAVID, the GO-BP FAT term is used to report enrichment results, as it “attempts to filter the broadest terms so that they do not overshadow the more specific terms” [50]. The biological process “translation” is the only GO term that is detected as enriched (with FDR = 8.9*e* − 19) for the genes appearing in Cluster 1. The genes involved in both clusters with significant GO terms are listed in Table 2. 

There are a quite few genes involved in multiple (independent) processes. For example, we find that the processes “amino acid activation” and “tRNA metabolic process” share 19 genes in common, while these two processes have no ancestor—child relationship. There is only one gene in Cluster 2 that is not in Cluster 1. This gene is involved in the “glycolysis.” As a point of future research, we noticed that the phase information is often used to cluster cell cycle gene expression profiles [35, 44, 51]. As such, a nature evolution of our approach is to employ phase information in clustering (see Section 5). 

## 5. Discussion and Conclusion

 When the application is gene expression, methods from signal processing have proven successful in decomposing the nature of complicated time series that contain multiple component signals. A two-step cluster validation is proposed to statistically determine the optimal number of clusters and to select the statistically significant clusters. To our knowledge, there are no clustering approaches that provide unique gene sets at different time points (i.e., genes in the same cluster may have different starting and ending points, and even have different time durations in the cluster). A simulation study demonstrates the benefits of our approach by showing that it is able to capture meaningful signals and separate them, even for very noisy data. Finally, we understand and acknowledge that it would be useful and encouraging to compare our method with other existing methods. Unfortunately, this is not possible simply because no information about time is contained in the clusters that are obtained by other methods. Time information is the critical component for both determining the clusters obtained by our method and calculating the “dynamic index” formula which measures the clustering performance.

The proposed method focuses on clustering periodic time series by considering the spectral frequencies that are decomposed and extracted from periodic data. Beyond the spectral frequency, phase information is obtained as well in the signal decomposition. In fact, clustering only the spectral frequency of time series may not be sufficient to understand very complicated biological processes. In other words, even though two genes involved in the same biological process have the same spectral frequencies, they may play different roles in the process. For example, one gene may serve as a regulator for the other. Studying the phase relationship between genes may help understand such regulation and is a point of future research. Further, consider two genes that participate in different phases of a cellular process. A phase study is certainly necessary after clustering the spectral frequencies. It is worth noting that we cannot change the order of studies, (i.e., perform a phase study first followed by a frequency study) since genes with different spectral frequency must belong to different biological processes, and genes with different phases may or may not belong to the same process. Therefore, within each cluster of spectral frequency, genes can be subclustered according to their component phases so that the gene relationship may be revealed in greater detail [34]. 

Genes involved in multiple biological processes (simultaneously) may play a major role in one process while playing a minor role in another process. The importance of a gene in multiple processes has potential for further investigation. Since the energy of a time series is proportional to its amplitude squared, the importance of a gene in a process can be measured using the squared amplitude of its corresponding component. Based on this, and due to the fact that genes may participate in different processes at different time, the dynamic importance of genes in biological processes can be established. As a point of future research, if three features of periodic time series, namely, spectral frequency, phase shift, and amplitude, are all included, as well as the time information of components, the complex dynamic biological processes may be better understood [34].

Because the proposed dynamic clustering process is time dependent, an appreciation for the number of time points that are recommended for the method is a necessary discussion. Since the main feature of a periodic time series is spectral frequency, frequency detection is highly reliant on the sampling rate. Therefore, the minimal or recommended number of time points is related to the nature of biological processes/clusters in which the genes are involved. According to the Sampling Theorem [52], a signal can be exactly recovered if the sampling rate is greater than twice the signal frequency. Thus, if a signal has 5 cycles in one hour duration, the sampling rate must be more than 20 points in one hour. In other words, if the sampling rate is small, then a signal with high frequency cannot be detected. 

Finally, since spectral frequencies are extracted from periodic time series, the time points occur at equally spaced intervals. For periodic time series with unevenly spaced points, evenly spaced time points can be artificially created by imputing missing data. Thus, our proposed approach is applicable, but some information may be lost due to data imputation. Further, since the proposed two-stage approach (i.e., data preparation and dynamic clustering) is designed for periodic data, for data that are not periodic, the signal decomposition approach is not applicable in the data preparation step. However, if some other characteristic can be defined and extracted from the nonperiodic time series, the second step of the proposed approach remains applicable. 

 Dynamic clustering is a two-step cluster validation that is able to differentiate meaningful clusters from noisy clusters. The results from our approach provide insight into the dynamic association among time-limited coexpressed genes that might otherwise go undetected by current clustering approaches. Clustering and gene network inference are both known to help in predicting the biological functions of genes or unraveling the mechanisms involved in biological processes. Usually clustering and gene network methods are developed independently. Specifically, in the gene networks, the challenge is to deal with a large number of genes, but when clustering, the clusters are assumed independent. In actuality, gene network procedures and gene clustering procedures cover each other's shortcomings [53]. As such, the proposed dynamic clustering has great potential for inferring gene networks, in particular, for exploring networks at the level of gene clusters. Although the proposed method is motivated by and explained in the context of time series microarray data, it is a general method that is applicable to any periodic phenomena, including but not limited to seasonal data in marketing research, meteorology, and astronomy. 

## Figures and Tables

**Table 1:** Dynamic indices for simulated data (1000 datasets) at three different noise levels.

	Noise level		
	0.5	1.0	1.5
Average of dynamic index	0.999	0.910	0.653
St. dev. of dynamic index	8*e* − 4	0.012	0.024

**Table 2:** *P. falciparum* genes appearing in both clusters and those having significant gene ontology (GO) terms.

GO id	GO name	FDR
GO:0006412	Translation	1.55*E* − 13
GO:0006260	DNA replication	1.12*E* − 07
GO:0006418	tRNA aminoacylation for protein translation	7.87*E* − 04
GO:0043038	Amino acid activation	7.87*E* − 04
GO:0043039	tRNA aminoacylation	7.87*E* − 04
GO:0006399	tRNA metabolic process	0.00962
